# Synthesis of hapten, generation of specific polyclonal antibody and development of ELISA with high sensitivity for therapeutic monitoring of crizotinib

**DOI:** 10.1371/journal.pone.0212048

**Published:** 2019-02-11

**Authors:** Mona M. Al-Shehri, Adel S. El-Azab, Manal A. El-Gendy, Mohammed A. Hamidaddin, Ibrahim A. Darwish

**Affiliations:** 1 Department of Pharmaceutical Chemistry, College of Pharmacy, King Saud University, Riyadh, Saudi Arabia; 2 Department of Medicinal and Analytical Chemistry, Faculty of Pharmacy, Sana’a University, Sana’a, Yemen; Kinki Daigaku, JAPAN

## Abstract

Crizotinib (CZT) is a potent drug used for treatment of non-small cell lung cancer (NSCLC); however, its circulating concentration variability has been associated with acquired resistance and toxicity, restricting the success of cancer treatment. As such, the development of an assay that monitors CZT plasma concentrations in patients is a valuable tool in cancer treatment. In this study, a hapten of CZT was synthesized by introducing the acetohydrazide moiety as a spacer into the chemical structure of CZT. The chemical structure of the CZT acetohydrazide (hapten) was confirmed by mass, ^1^H-, and ^13^C-NMR spectrometric techniques. The hapten was coupled to each of bovine serum albumin (BSA) and keyhole limpet hemocyanin (KLH) proteins by ethyl-3-(3-dimethylaminopropyl) carbodiimide as a coupling reagent. CZT-KLH conjugate was used for immunization and generation of a polyclonal antibody recognizing CZT with high affinity (IC_50_ = 0.5 ng/mL). The polyclonal antibody was used in the development of an ELISA for determination of CZT. The ELISA involved a competitive binding reaction between CZT, in its samples, and immobilized CZT-BSA conjugate for the binding sites on a limited amount of the anti-CZT antibody. The assay limit of detection was 0.03 ng/mL and the working range was 0.05 − 24 ng/mL. Analytical recovery of CZT from spiked plasma was 101.98 ± 2.99%. The precisions of the assay were satisfactory; RSD was 3.2 − 6.5% and 4.8 − 8.2%, for the intra- and inter-assay precision, respectively. The assay is superior to all the existing chromatographic methods for CZT in terms of its procedure simplicity, convenience, and does not require treatment of plasma samples prior to the analysis. The proposed ELISA is anticipated to effectively contribute to the therapeutic monitoring of CZT in clinical settings.

## Introduction

Lung cancer is the most common cancer in terms of both incidence and mortality in men and women [1]. In 2016, the estimated new cases and deaths from lung cancer in the United States were 224,390 and 158,080, respectively [2]. According to the latest World Health Organization (WHO) data published in 2017, lung cancers deaths in Saudi Arabia reached 906 which represent 0.93% of the total deaths. The main types of lung cancers are small-cell lung cancer and non-small cell lung cancer (NSCLC). NSCLC accounts for ~ 85% of all lung cancers. These cancer cells grow quickly and spread early in the course of the disease [3]. Crizotinib (CZT) is a potent small-molecule drug of the tyrosine kinase inhibitors group drug used for treatment of NSCLC [4]. CZT is chemically named as 3-[(1R)-1-(2,6-dichloro-3-fluorophenyl) ethoxy]-5-(1-piperidin-4-ylpyrazol-4-yl)pyridine-2-amine. It is a potent small-molecule drug of the tyrosine kinase inhibitors group [4]. CZT has demonstrated high response rates in non-small cell lung cancer (NSCLC) patients carrying anaplastic lymphoma kinase (ALK (fusion gene [5]. This gene results in constitutive kinase activity that contributes to carcinogenesis and drive the malignant phenotype [6,7]. On August 26, 2011, the Food and Drug Administration (FDA) has granted accelerated approval for CZT-containing capsules (under the trade name of Xalkori capsules made by Pfizer, Inc.) for the treatment of advanced local or metastatic NSCLC. This accelerated approval was based on successful clinical multi-center studies on CZT [6]. However, the determination of CZT in biological fluids for the purpose of its therapeutic drug monitoring (TDM) is still very important to ensure its effective and safe therapy. TDM of CZT is seriously important because it has shown variability in its circulating concentrations among patients during therapy of patients with NSCLC, favoring the selection of resistant cellular clones in case of sub-therapeutic drug exposure, or increasing the risk of adverse drug reactions at excessive plasma levels [8–10].

Extensive literature survey showed that CZT has been determined in biological fluids by liquid chromatography (LC) with fluorescence [11] and mass (MS) [12–18] detectors. LC-MS is a valuable tool; however, its high cost and instrumentation complexity limit its routine application in clinical laboratories. Immunoassays (e.g. ELISA) are more preferable alternative techniques in the field of clinical analysis [19]. This was attributed to the facts that they are specific for the analyte, they usually do not require pretreatment for the specimens of complex matrix (e.g. plasma, urine, etc.), they have high analytical throughputs thus are suited for clinical setting processing large number of samples, and the analysis by these assays is not expensive. These reasons were behind our interest in the development of immunoassay for CZT. The present study describes, for the first time, the synthesis of acetohydrazide derivative as hapten for CZT with 4-atoms spacer and is able to directly conjugated to protein carriers, preparation of a polyclonal antibody that able to recognize CZT with high affinity, and establishment of an ELISA for determination of CZT in plasma samples for the purpose of its TDM.

## Experimental

### Instruments

Microplate /cuvette reader (Spectramax M5: Molecular Devices, California, USA). Automatic microplate strip washer (MW-12A: Bio-Medical Electronics Co. Ltd., Shenzhen, China). HPLC apparatus equipped with Waters 1525 binary HPLC pump, Waters 2707 autosampler and Waters 2489 UV-visible detector (Waters Corporation, Milford, Massachusetts, USA). UV-visible spectrophotometer (V-530: JASCO, Tokyo, Japan). Mass spectrometer (Varian TQ 320 GC/MS/MS: Varian, Palo Alto, USA). FT-IR spectrophotometer (Shimadzu, Osaka, Japan). Bruker NMR spectrometer (Bruker Corporation, Bruker Daltonik GmbH, Bremen, Germany). Milli-Q water purification system (Labo, Millipore Ltd., Bedford, USA).

### Materials

Crizotinib (Shanghai Haoyuan Chemexpress Co., Ltd. Shanghai, China) used as received; its purity was > 99%. Bovine serum albumin (BSA), 3,3`,5,5`-tetramethyl-benzidine, peroxidase substrate solution (TMB), *N*-hydroxysuccinamide (NHS) and 1-ethyl-3-(3-dimethylaminopropyl carbodiimide HCl (EDC) were purchased from Sigma Chemicals Co. (St. Louis, USA). Keyhole limpet hemocyanin (KLH) was purchased from Novabiochem Co. (La Jolla, USA). Freund’s adjuvants (complete and incomplete), dialysis tubes cell membrane, and goat anti-mouse IgG-horseradish peroxidase conjugate (HRP-IgG, catalogue number: A5278) were obtained from Sigma-Aldrich (St. Louis, USA). BCA protein assay kit was a product of Pierce Chemical Co. (Rockford, USA). EIA/RIA high-binding flat bottom polystyrene 96-microwell plates) were obtained from Corning/Costar Inc., Corning (New York, USA). Waters C18 analytical HPLC column (150 mm × 4.6 mm, 5 μm) manufactured by Altmann Analytik GmbH & Co. (Deutschland, Germany). All other chemicals and solvents used throughout the work were of analytical grade.

### Procedures

#### Synthesis of CZT hapten (CZT acetohydrazide)

An amount of CZT (450 mg equivalent to 1 mmol) was dissolved in 15 mL acetone. To this solution, 167 mg (equivalent to 1 mmol) of ethyl 2-bromoacetate and 277 mg (equivalent to 2 mmol) of potassium carbonate anhydrous were added. The contents were mixed and the mixture was stirred for 12 h at room temperature (25 ± 2°C). The produced solid material was isolated by filtration. This intermediate solid was washed with water and dried. The dried solid intermediate (536 mg) was dissolved in 10 mL of absolute methanol and 75 mg of hydrazine hydrate was added. The reaction mixture was stirred for 24 h at room temperature. The reaction was monitored by HPLC system; the chromatographic conditions were: the mobile phase consisted of acetonitrile and 50 mM PB of pH 5 (1:1, v/v). Elution was performed isocratically at a flow rate of 2 mL/min and column temperature was maintained at room temperature (25 ± 2 ^o^C). The injection volume was 20 μL, and the detection wavelength was 254 nm. After the reaction completion, the reaction mixture was filtered and the solid obtained was dried as acetohydrazide derivative of CZT (CZT hapten). The purified CZT hapten was subjected to the analysis by different spectroscopic techniques in order to confirm its chemical structure. These techniques were MS, FT-IR, ^1^H-NMR operating at 500 mHz, ^13^C-NMR operating at 125.76 MHz. NMR spectra were scanned in DMSO-d6 and the chemical shifts were expressed in ppm, relative to tetramethylsilane (TMS) as an internal standard. In order to confirm the exchangeable protons, deuterium oxide (D_2_O) was added.

#### Preparation of CZT-protein conjugates

A quantity (50 mg) of each of BSA and KLH protein was dissolved in 5 mL of phosphate buffer (PB) solution (50 mM, pH 7.2) producing protein solutions. Solution of hapten (CZT acetohydrazide) was prepared by dissolving 50 mg of the hapten material in 5 mL of PB (50 mM, pH 7.2). To each protein solution, 100 mg of EDC and 100 mg of NHS were added and the pH of the reaction mixture was rapidly adjusted to 5–5.5 with 0.01 M HCl. After 5 min, hapten solution was added to the protein solution and the pH of the mixture was rapidly adjusted to pH 6.4 and kept constant for further 90 min. The reactions were allowed to proceed in dark at 4°C for overnight. The reaction mixture was subjected to dialysis to remove any remaining unreacted hapten molecules from the CZT-protein conjugates. After dialysis, the products (CZT-protein conjugates) were collected and subjected to analysis as proteins by protein assay kit and spectral analysis by UV-spectrophotometry.

#### Characterization of CZT-protein conjugates

The protein contents of BSA and KLH conjugates of CZT were estimated by BCA protein assay kit, according to the procedure described by manufacture of the kit. Confirmation of conjugation of CZT to protein was conducted by UV-absorption spectral analysis. Solutions of CZT hapten, CZT-BSA, BSA, CZT-KLH and KLH were prepared in phosphate buffer saline (PBS) solution (50 mM, pH 7.2). These concentrations were 0.1, 0.6, 0.6, 1.6, and 1.6 mg/mL, respectively. The UV-spectra of these solutions were recorded in the range of 200–400 nm. The spectra of the conjugates were compared with those of the unconjugated proteins that have been generated using the same concentrations under the same pH conditions. The shape of these spectra and molar extinction coefficients were examined for confirmation of the successful conjugation of CZT hapten with the proteins.

#### Immunization of animals and preparation of polyclonal antibody

The immunization study was approved by the Institutional Ethics Committee of King Saud University. The immunogen solution (1 mg/mL of CZT-KLH conjugate prepared in PBS) was mixed with an equal volume of complete Freund’s adjuvant and the mixture was emulsified. Four female 8-weeks old BALB/c mice were subjected to the immunization. Mice were kept in polycarbonate plastic cages containing wood shavings as bedding. The food and water were freely available all the housing time. Anesthesia and/or analgesia have not been used in the immunization. The immunization has been conducted as per the procedures described by Darwish *et al*. [20]. Briefly; each mouse was injected intraperitoneally with 100 μL of the CZT-KLH conjugate emulsified in Freund’s complete adjuvant. The animals were subjected to 6 boosting immunizations every 2 − 3 weeks interval. From the immunized animals, total blood were collected, diluted in PBS and the sera (supernatants) were isolated after centrifugation the blood samples at 10,000 g at 4°C for 10 min. The sera were analyzed for their anti-CZT antibody contents using the procedures described by Darwish *et al*. [21]. The antibody response in each mouse was determined by analysis of the collected antisera by direct ELISA described by Darwish *et al*. [21]. The mouse serum that showed the highest avidity to CZT (lower IC_50_ value) was used as the crude polyclonal antibody for CZT. In the end of experiments animals were sacrified by exposure to CO_2_ gas according to the Guidelines for euthanasia of rodent fetuses and neonates [22].

#### ELISA procedures, data acquisition and analysis

Fifty microliters of CZT-BSA conjugate solution (2 μg/mL prepared in PB) was dispensed into each well of the assay plate and the coating of the conjugate was allowed to proceed by incubating the assay plate at 37°C for 2 h. The plate was subjected to washing three times with washing buffer solution using microplate washer. The wells were blocked with 100 μL of the blocking buffer solution (1% BSA in PBS) and incubated for 1 h at 37°C, and then washing the plate three times with the washing buffer solution. Samples of standard solution of CZT (0.02–24 ng/mL) or CZT-spiked plasma samples (5 μL plasma diluted to 200 μL with PBS) were prepared. Fifty microliters of these samples were mixed with 50 μL of anti-CZT antibody (diluted 160-fold in PBS), and 50 μL of the mixture was dispensed into each well of the assay plate. After incubation for 2 h at 37°C, the plate was washed, and 50 μL of secondary antibody (HRP-IgG; diluted 1:1000 in PBS) was added to each well. The binding of HRP-IgG was allowed to proceed by incubation for 2 h at 37°C, and then the plate was washed as previously described. TMB solution (50 μL) was added and the plate was incubated for 30 min at 37°C for color development 50 μL of 2 N HCl to each well to stop the reaction. The absorbances were measured at 450 nm using the microplate absorbance reader.

The data was adquired by Spectramax software (Spectramax M5: Molecular Devices, California, USA), and transformed to a Slide Write software, version 5.011 (Advanced Graphics Software Inc., USA) for fitting using the 4-parameter equation in the software and creating the calibration curve, from which the concentrations of CZT in the samples were determined

## Results and discussion

### Synthesis of hapten and confirmation of its structure

**Fig 1:**
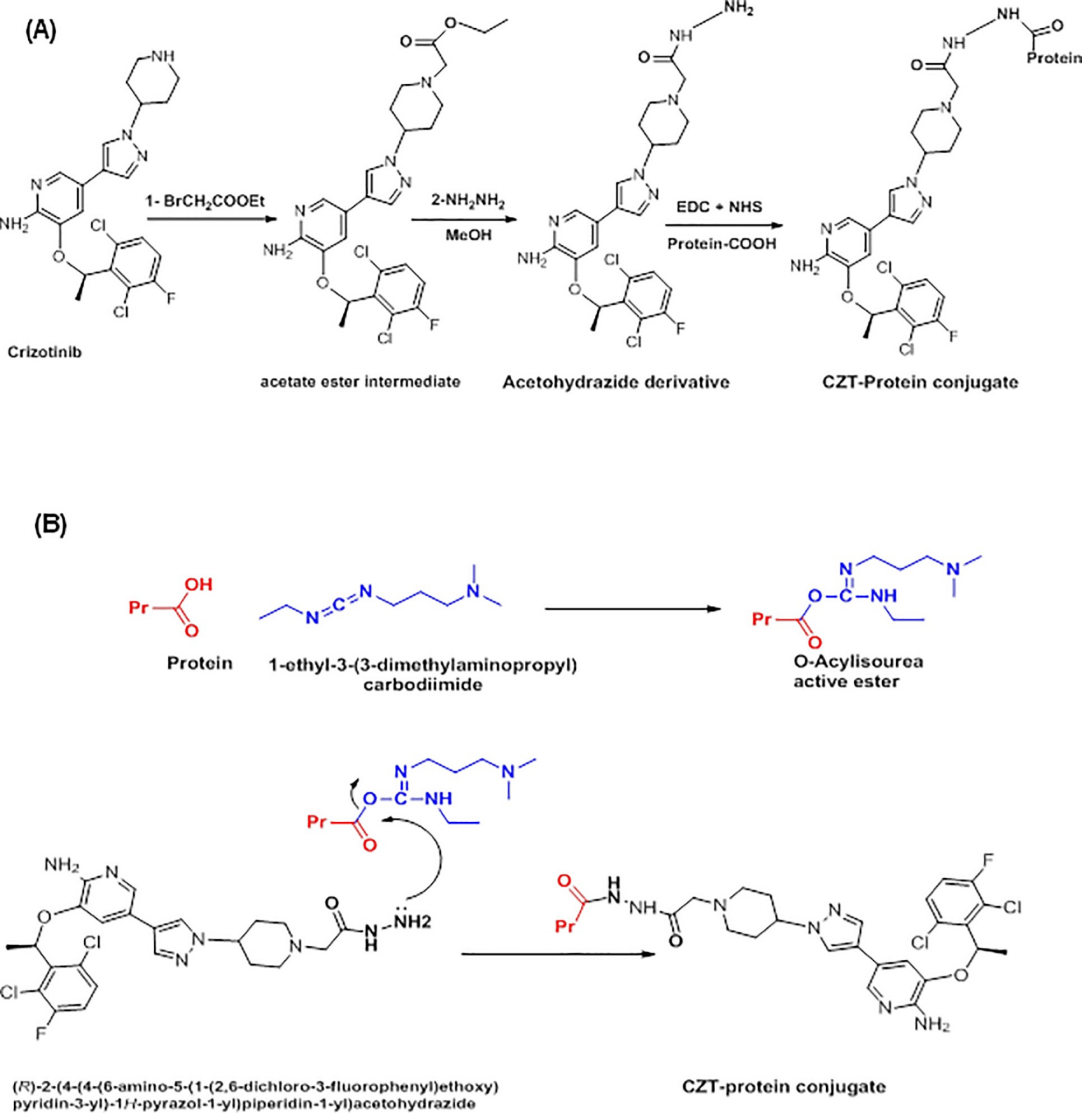
Reaction pathway for synthesis acetohydrazide derivative of CZT and its coupling with proteins (A) and the coupling reaction mechanism (B). Protein-COOH is the BSA and KLH, EDC is 1-ethyl-3-(3-dimethyl-aminopropyl carbodiimide hydrochloride and NHS is *N*-hydroxysuccinimide.

CZT is a small molecule drug; therefore its nature is not immunogenic and accordingly is unable to induce immune response and produce antibodies. In order to convert CZT to immunogenic molecule, it should be linked to some immunogenic macromolecules such as carrier proteins [21]. CZT could be linked directly to proteins by formation of its diazonium salt involving the aromatic amino group in its chemical structure followed by coupling the diazonium salt to the proteins. However, the presence of a “spacer group” between the small molecule and the protein usually leads to induction of antibodies with improved specificity of the small analyte molecule linked to the immunogenic protein [23]. Therefore, it was required to modify the structure of CZT by a synthetic reaction to introduce a spacing reactive functional group that enables its coupling with carrier proteins. This modification was achieved by synthesis of the acetohydrazide derivative of CZT. The synthesis was conducted in two steps (Fig 1A); the first one is the reaction of CZT with ethylbromoacetate to form acetate ester intermediate. The second step is the reaction of acetate ester intermediate with hydrazine hydrate to produce acetohydrazide derivative of CZT (CZT hapten). The reaction was monitored by HPLC with UV detection at 254 nm, and the retention times were 3.616, 3.991, and 2.548 min for starting CZT material, the acetate ester intermediate, and the reaction product (CZT acetohydrazide derivative), respectively (S1A Fig). The yields and puritiesof each step was > 98%. The chemical structure of the CZT acetohydrazide was ellucidated by mass spectrometry that gave a molecular weight of 522; [M+1].

The FT-IR (KBr, cm^-1^) analysis of the product (Fig 1B) revealed the presence of peaks at 1656 (C = O), 3481, 3381, and 3323 (NH). The ^1^H -NMR (S2 Fig) and ^13^C-NMR (S3 Fig.) spectral data are presented in Tables 1 and 2, respectively. These spectral data were compared with those of CZT standard material. The appearance of carbonyl group of acetohydrazide derivative of CZT was demonstrated (S3 Fig). Besides, the difference was obvious between CZT and its synthesized CZT acetohydrazide at the up field region of the spectra confirming the presence of aliphatic moiety in the side chain of the synthesized acetohydrazide derivative of CZT.

**Table 1:** ^1^H-NMR spectral data of acetohydrazide derivative of CZT.

Signal	Location (δ)	Shape	Integration	Correspondences
1	1.06	s	6H	2CH_3_
2	1.49–1.50	t, J = 2.8, 2.1 Hz	2H	CH_2_
3	1.85	d, J = 6.5 Hz,	3H	O(CH)CH_3_)
4	2.13−2.06	m	4H	Aliphatic
5	2.37	t, J = 11.0, 11.5 Hz	2H	Aliphatic
6	2.99	d, J = 11.5 Hz	2H	Aliphatic
7	3.14	s	2H	CH_2_CONHNH_2_
8	4.13−4.09	m	1H	Aliphatic
9	4.88	s	2H	NH_2_
10	6.08	t, J = 6.0, 6.5 Hz	1H	O(CH)CH_3_)
11	6.87	s	1H	Aromatic
12	7.05	t, J = 8.0, 8.5 Hz	1H	Aromatic
13	7.32−7.28	m	1H	Aromatic
14	7.49–7.76	s	1H	Aromatic

**Table 2:** ^13^C-NMR spectral data of acetohydrazide derivative of CZT.

Signal	Location (δ)	Correspondences
1	18.90	CH_3_
2	32.55	Aliphatic
3	53.17	Aliphatic
4	58.61	Aliphatic
5	60.57	CH_2_CONHNH_2_
6	72.44	O(CH)CH_3_
7	116.63	Aromatic
8	119.03	Aromatic
9	119.97	Aromatic
10	121.96	Aromatic
11	122.12	Aromatic
12	122.78	Aromatic
13	128.96	Aromatic
14	135.48	Aromatic
15	135.87	Aromatic
16	136.94	Aromatic
17	139.84	Aromatic
18	149.01	Aromatic
19	170.37	CO

### Preparation and characterization for CZT-protein conjugates

Acetohydrazide derivative of CZT (hapten) was successfully linked to each of bovine serum albumin (BSA) and keyhole limpet hemocyanin (KLH) proteins by carbodiimide coupling reagent [21]; the coupling reaction is described in Fig 1. To confirm the linking of CZT hapten residues to the protein molecules and formation of the CZT-protein conjugates, the potential conjugates were analyzed by UV-spectrophotometry and the generated spectra were compared with those of the hapten and the unconjugated proteins generated under the same conditions by the same concentrations (S4 Fig). The protein conjugates exhibited higher light absorption than the unconjugated protein did at their maximum absorption wavelengths. The higher absorbances observed in the spectra of protein conjugates over those of the same concentrations of proteins were evident for the successful conjugation of the chromophoric CZT hapten molecules with the proteins.

### Generation of anti-CZT polyclonal antibody and its characterization

**Fig 2:**
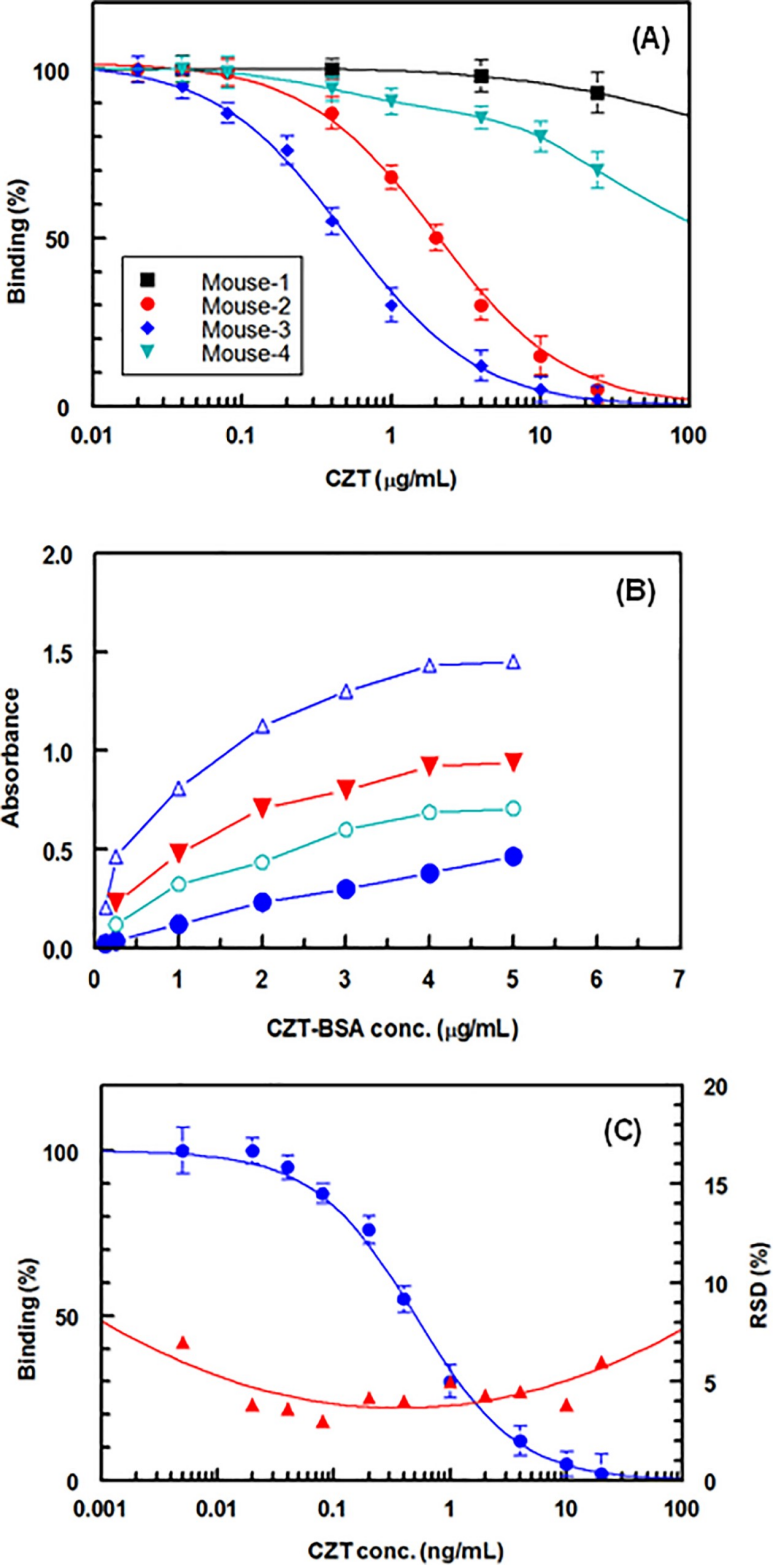
Panel (A): affinity of antisera from mice (1–4) for CZT, panel (B): checkerboard titration for CZT-BSA versus anti-CZT antibody. Varying concentrations of CZT-BSA conjugate (0.15 − 5 μg/mL) were coated onto the microwell plates and varying dilutions of the anti-CZT antibody were allowed to bind to the coated CZT-BSA. Dilutions of anti-CZT antibody were 20 (●), 40 (○), 80 (▼), 160 (△) folds. Panel (C): calibration curve (●) and precision profile (▲) of the proposed ELISA for CZT.

KLH protein has higher immunogenic properties than BSA [22], thus CZT-KLH conjugate was as immunogen for generation of anti-CZT polyclonal antibody and the CZT-BSA conjugate was used solid-phase immobilized reagent in the assay. Four mice were immunized with CZT-KLH and the immune response was tested by analyzing the collected antisera by direct and competitive enzyme immunoassays described by Darwish *et al*. [20]. The parameters that have been considered in testing the evoked antisera were their reactivity to the immobilized CZT-BSA, and their affinity to CZT itself. It was found that antisera collected from mouse No. 3 was the most appropriate one as it gave the highest affinity for CZT (Fig 2A); therefore, its total antisera was collected and used as the anti-CZT polyclonal antibody in the development of the ELISA described herein for CZT.

### The proposed ELISA and optimizing of its conditions

**Fig 3:**
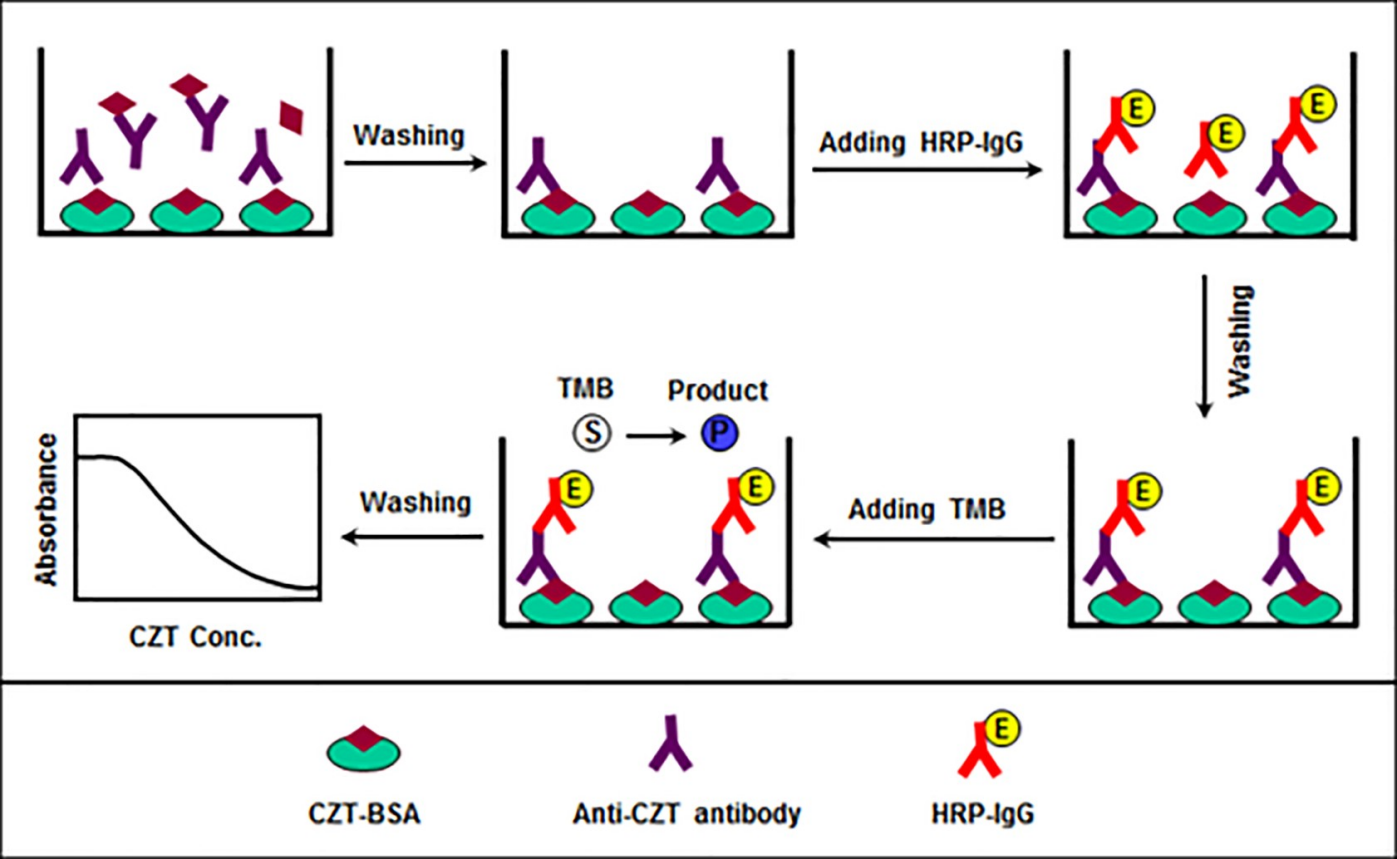
Schematic diagram for the proposed ELISA for CZT.

The proposed ELISA is an antibody-capture ELISA; Fig 3 illustrates the features of this assay. In this ELISA, CZT-BSA was coated onto the inner surface of microwells of an assay plate and the plate was used as a solid support. CZT was mixed with a pre-determined limited amount of the anti-CZT polyclonal antibody, a 50 μL of the mixed solution was transferred into the microwells that are already coated with CZT-BSA, and the competitive binding reaction was allowed to proceed. The amount of anti-CZT antibody bound to the plate wells was then quantified by HRP-IgG and TMB as a chromogenic substrate for the peroxidase enzyme. The intensity of the produced color was inversely proportional to the concentration of CZT in its sample solution. The experimental conditions play an important role in the development and analytical performance of ELISA for any particular analyte. Based on previous studies [24–26], the solutions of the immunoanalytical reagents involved in this study were prepared in PBS of pH 7.4. As well, the HRP-IgG secondary antibody at a dilution of 1:1000 was used for generating the signal with TMB solution. The following sections describe the optimization of the conditions affecting the analytical performance of the proposed ELISA.

### Selection of CZT-BSA concentration and antibody dilution

As the proposed ELISA involves a competitive binding between the free CZT (in the sample solution) and the immobilized CZT (CZT-BSA coated onto the assay plate) for the available binding sites on the anti-CZT antibody, the amount of antibody used in the assay should be limited for the immobilized CZT-BSA conjugate. These conditions would provide an effective competition and attain high sensitive assay. In order to fulfil these requirements, checkerboard titration [27] for different dilutions for antibody versus varying concentrations of CZT-BSA conjugate coated onto the assay plate was conducted. The absorbances were measured and plotted as a function of the concentrations of CZT-BSA conjugate at different corresponding anti-CZT antibody dilutions (Fig 2B). It is obvious that all the dilutions of anti-CZT antibody were limited overall the CZT-BSA concentrations. Accordingly, competitive assays were conducted and it was found that the highest assay sensitivity (lowest IC_50_) was attained when the CZT-BSA conjugate was 2 μg/mL and the dilution of anti-CZT antibody was 160 folds. Therefore, these conditions were selected as optimum for all the subsequent experiments.

### Coating of CZT-BSA onto the assay plate and blocking the plate wells

In order to select the optimum coating buffer required for coating of CZT-BSA conjugate to the microwells, the conjugate was prepared in PB solution of pH 7.4 and compared with carbonate/bicarbonate buffer solution of pH 9.6. The results revealed that using PB solution as coating buffer was better than carbonate/bicarbonate. A set of experiments were conducted to establish the temperature and time of incubation required for optimum coating of the CZT-BSA onto the microwells, 50 μL of the CZT-BSA solution (2 μg/mL) was dispensed into each well of the assay plates. The plates were incubated for overnight at 4°C and for 2 h at 37°C, and then the plates were manipulated as usual. It was found that the coating of CZT-BSA conjugate for 2 h at 37°C was better than coating for overnight at 4°C. Longer incubation time at 37°C did not affect the coating efficiency. After coating of CZT-BSA conjugates onto the microwells, the remaining binding sites that were available on the surface of the microwells were blocked by 100 μL of BSA solution (0.5%, w/v, prepared in PBS) incubated at 37°C for 30 min.

### Competitive binding reaction and color development

Experiments were conducted to establish the proper time required for attaining the equilibrium of competitive binding reaction between CZT and its antibody and achieving the maximum assay sensitivity, the reaction was allowed to proceed for varying times (0.5–2 h). The results revealed that the minimum time required for equilibrium (indicated by obtaining constant reproducible IC_50_ values) was 2 h at 37°C, and longer time did not improve the IC_50_ values.

In order to select the most appropriate dilution of HRP-IgG, different dilutions (1:1000; 1:2000; 1:4000) were tested. The best results were obtained using the of 1:1000 dilution, giving the highest readings. As well, the binding of HRP-IgG at this dilution at room temperature (25 ± 2°C) was superior to the 37°C condition and the optimum time for its binding was 60 min. The optimization for color developing revealed that the incubation of TMB at 37°C for 30 min was the optimum conditions. Under these conditions, the absorbance values were 1.3 ± 0.12; however, the absorbances using the anti-CZT antibody and HRP-IgG alone (negative control) were 0.08 ± 0.05.

A summary for the optimization of conditions for development of ELISA for CZT is given in Table 3.

**Table 3:** Summary for the optimum conditions of ELISA for CZT.

Studied condition	Optimum
Coated CZT-BSA conc. (μg/mL)	2
Coating time (h) / temperature (°C)	2 / 37
BSA conc. (%, w/v) used for blocking	0.5
Blocking time (h) / temperature (°C)	30 / 37
Competitive binding reaction time (h) / temperature (°C)	2 / 37
HRP-IgG (dilution)	1:1000
Binding of HRP-IgG: time (min) / temperature (°C)	60 / 25
HRP/TMB color reaction: time (min) / temperature (°C)	30 / 37
Reagent for stopping the color reaction	2 N HCl
Measured color (wavelength, nm)	Yellow (450)

### Validation of ELISA for CZT

#### Calibration curve, its precision profile and assay sensitivity

The calibration curve of the proposed ELISA for CZT was constructed by plotting the binding (as pecentages) as a function of their corresponding CZT concentrations (Fig 2C). The CZT concentrations used for generating the curve was in the range of 0.02–24 ng/mL. The data analysis of the curve gave high correlation coefficient (r = 0.9992) upon their fitting on the four-parameter equation. The limit of detection (LOD) and limit of quantitation (LOQ) of the ELISA for CZT were determined as per the guidelines [28] and the LOD and LOQ were found to be 0.03 and 0.05 ng/mL, respectively. This sensitivity is adequate for the quantitation of CZT in plasma samples without any need for pre-concentration of CZT in the samples prior to their analysis as the reported level of CZT in plasma was 99.6 ng/mL [29].

#### Assay precisions

For assessement of the intra- and inter-assay precisions, three levels of CZT concentration were used; these levels were low, medium, and high (0.1, 0.5, and 2 ng/mL, respectively). For assessement of the intra-assay precision, 8 replicates of the same sample were analyzed by the proposed ELISA as a batch in a single assay run. For assessement of the inter-assay precision, duplicates of the same samples were analyzed in 4 separate assay runs. The results revealed the acceptable precisions of the ELISA as per the requirements of immunoassay validation guidelines [30]. The relative standard deviation (RSD) for the intra-assay precisions were 3.2–6.5% (Table 4). For the inter-assay precisions, RSD values were 4.5–8.2%. These acceptable precisions of the proposed ELISA was attributed to two facts. The first fact is the uniformity of coated CZT-BSA quantity from well-to-well of the assay plate, and the second fact is the steps of incubations involved in the assay were conducted at consistent temperature.

**Table 4:** Precisions of the proposed ELISA for CZT at different concentration levels.

Concentration (ng/mL)	Recovery (% ± RSD)	
	Intra-assay	Inter-assay
0.1	98.5 ± 6.5 ^a^	101.4 ± 8.2
0.5	102.4 ± 3.2	102.7 ± 4.5
2	103.7 ± 5.4	98.2 ± 6.4

^a^ Values are mean of recovery values of 8 determinations **±** relative standard deviation (RSD).

#### Accuracy and effect of plasma matrix

Accuracy of the assay was evaluated by recovery studies. Recovery was determined by spiking known CZT concentrations (0.13–4 ng/mL) to a CZT-free plasma, and the CZT-spiked plasma samples were diluted (5 μL plasma to 200 μL with PBS) and analyzed for their CZT contents by the proposed ELISA. The analytical recovery values were found to be 97.5–104.1% (Table 5). The mean analytical recovery value was 101.0 ± 2.99%. These acceptable recovery and RSD values [30], indicated the ability of the proposed ELISA for the accurate quantitation of CZT in plasma samples, as well as absence of any interferences from endogenous plasma sample’s components and/or matrix.

**Table 5:** Analytical recovery of CZT spiked in plasma samples.

Concentration of CZT (ng/mL)		Recovery (% ± RSD) ^a^
Spiked	Measured	
0.13	0.14 ± 0.006	104.0 ± 4.85
0.25	0.25 ± 0.011	98.0 ± 4.52
0.5	0.49 ± 0.020	97.5 ± 3.92
1.0	0.99 ± 0.046	99.5 ± 4.56
2	2.05 ± 0.097	102.6 ± 4.82
4	4.17 ± 0.020	104.1 ± 4.95
	Average 101.0 ± 2.99	

^a^ Values are mean of 3 determinations.

#### Comparison of the proposed ELISA with other techniques

The analytical performance of proposed ELISA was compared with the reported techniques for determination of CZT in human plasma, which are mostly liquid chromatography coupled with fluorescence detector [11] or tandem mass spectrometric detectors [12–15]. The comparison was in terms of the sensitivity, accuracy, precision, sample pretreatment and analytical throughput (Table 6). The sensitivity (expressed by LOQ) of the proposed ELISA is higher than all the reported chromatographic methods by 100 − 250 folds as the LOQ of ELISA was 0.02 ng/mL; whereas, it was 2–5 ng/mL for the chromatographic methods. The high sensitivity of ELISA was attributed to the use of antibody with high affinity and enzyme/substrate system for detection which usually offers extremely high sensitivity. The sensitivity of the proposed ELISA allowed use of small plasma samples from the patients, which is more convenient to the patients. The accuracy of the proposed ELISA was comparable with that of the reported chromatographic methods as the accuracy of ELISA was 101.0 ± 2.99%; whereas, it was in the range of 94.4–105.2% for the chromatographic methods. The proposed ELISA has better precision (lower RSD%) than the reported chromatographic methods. The better precision of ELISA was attributed to the uniformity of quantities of CZT-BSA coated onto the assay plate wells, conducting the steps of incubations at consistent temperatures, and absence of sample pretreatment procedures involved in the ELISA, rather than the chromatographic methods which mostly involved protein precipitation and/or sample extraction. The proposed ELISA depends on batch-analysis; however, the chromatographic methods depend on sequential-analysis making ELISA has higher throughput than chromatography. This feature is very beneficial in circumstances of pharmacokinetic studies whereas screening of large number of samples is required. These potential advantages of the proposed ELISA, in addition to the relatively low cost of the instruments, tools, or the reagents made ELISA potential alternative technique for the existing chromatographic techniques for bioanalysis of CZT.

**Table 6:** Comparison of ELISA with liquid chromatographic techniques for analysis of CZT in human plasma.

Technique	Pretreatment	Range (ng/mL)	LOQ (ng/mL)	Accuracy (Recovery %)	Precision (RSD, %)	Reference
ELISA	Dilution with PBS	0.02 − 24	0.02	101.0 ± 2.99	Intra-assay: 3.2–6.5; Inter-assay: 4.5–8.2	Present work
HPLC-FL	Protein precipitation	2–512	2	100.69 ±7.26	Intra-day: 5.69–10.05 Inter-day: 5.24–9.35	11
LC-MS/MS	Protein precipitation	5–1000	5	More than 95	Less than 15%	12
UPLC-MS/MS	Protein precipitation	5–500	5	99.7–105.2	Less than 15%	13
UPLC-MS/MS	Protein precipitation	2–2000	2	85.4–112.2	Less than 14%	14
LC-MS/MS	Dilution	5–5000	5	94.4–96.0	Less than 13%	15

## Conclusions

The present study is the first report describing the synthesis of acetohydrazide derivative for CZT as a hapten, preparation of a polyclonal antibody recognizing CZT with high affinity. This antibody was used in the development ELISA with high sensitivity for the accurate measurement of CZT in plasma samples with LOQ value of 0.05 ng/mL. These feastures of the assay made it potential tool in therapeutic monitoring of CZT during therapy of NSCLC patients in in clinical settings. This monitoring would be very helpful in providing a proper and safe therapy to patients. In addition, the assay protocol is simple and easy to be conducted as an analyst could analyze ~ 200 samples per day when used assay plates already coated with CZT-BSA conjugate and blocked with BSA. This add the convenience property to the proposed ELISA for application in clinical laboratories processing large number of samples. The proposed ELISA is superior to all the existing chromatographic methods for CZT in terms of its procedure simplicity, convenience, and does not require treatment of plasma samples prior to the analysis.

## Supporting information

S1 Fig HPLC chromatogram of acetohydrazide derivative of CZT (A) and its IR spectrum (B).
(DOCX)

S2 Fig ^1^H-NMR spectrum of acetohydrazide derivative of CZT. (DOCX)

S3 Fig ^13^C-NMR spectra of acetohydrazide derivative of CZT (A) and that of the standard CZT (B).
(DOCX)

S4 Fig UV-absorption spectral analysis for CZT-protein conjugates with KLH (A) and BSA (B). In panel A: concentrations were 0.1, 1.6, and 1.6 mg/mL for CZT hapten, KLH, and CZT-KLH, respectively. In panel B: concentrations were 0.1, 0.6, and 0.6 mg/mL for CZT hapten, BSA, and CZT-BSA, respectively.
(DOCX)
