# Supplementary figures and images for: Synthesis of hapten, generation of specific polyclonal antibody and development of ELISA with high sensitivity for therapeutic monitoring of crizotinib
Source: PLoS One. 2019 Feb 11;14(2):e0212048. doi: 10.1371/journal.pone.0212048 (PMC6370279; doi:10.1371/journal.pone.0212048)

**S1 Figure**


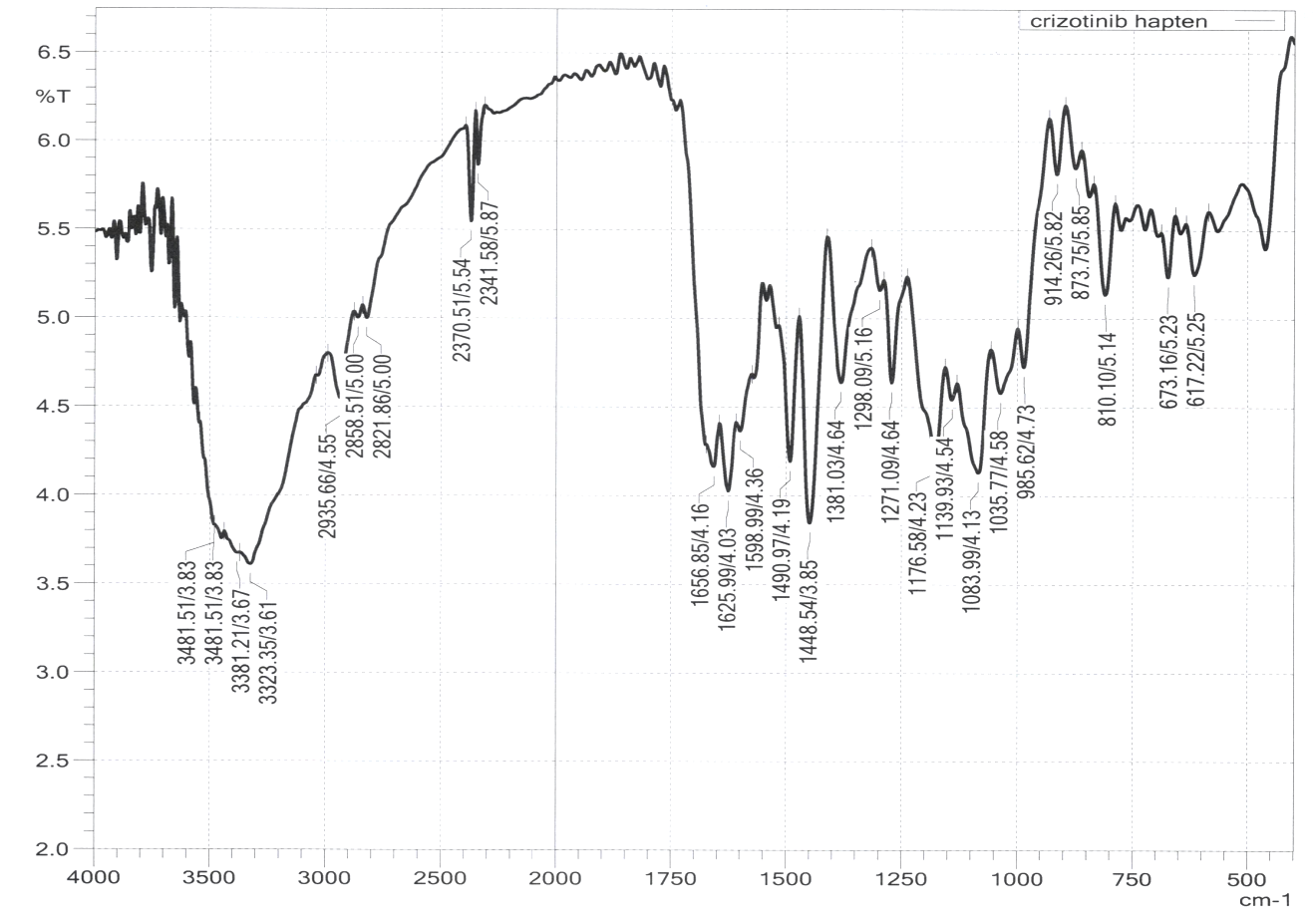

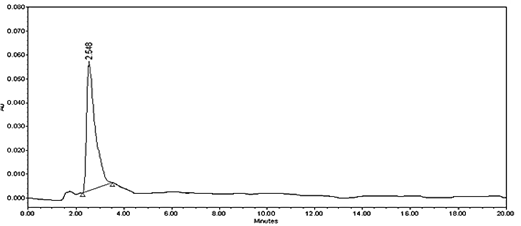


**(A)**

**(B)**

Supplement: S1 Fig — HPLC chromatogram of acetohydrazide derivative of CZT (A) and its IR spectrum (B). (DOCX) [file pone.0212048.s001.docx]

**S2 Figure**


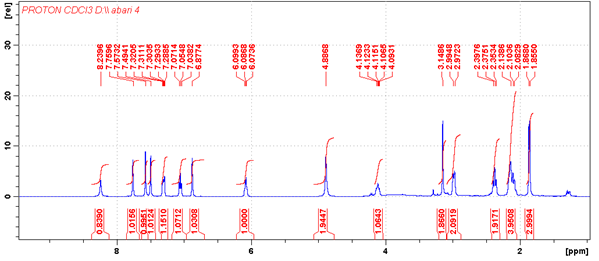

Supplement: S2 Fig — (DOCX) [file pone.0212048.s002.docx]

**S3 Figure**


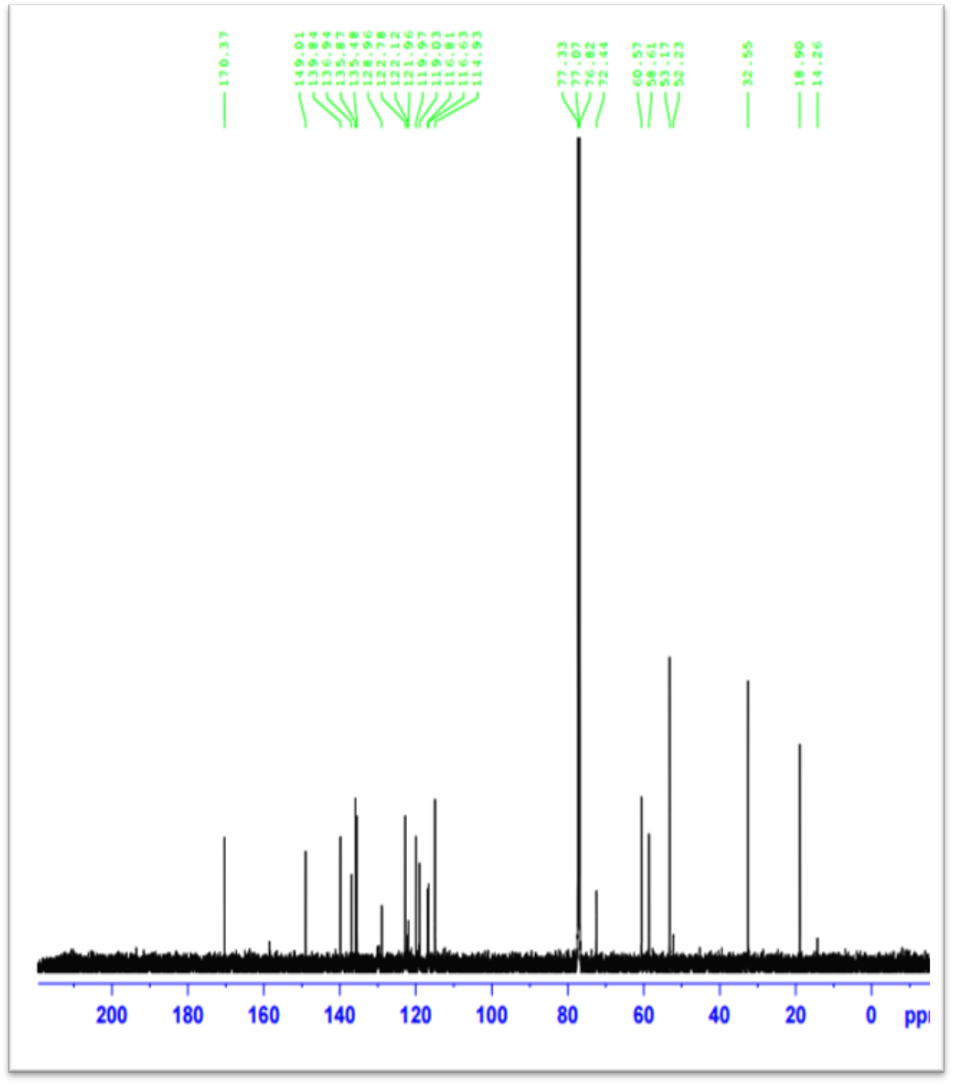


**(A)**


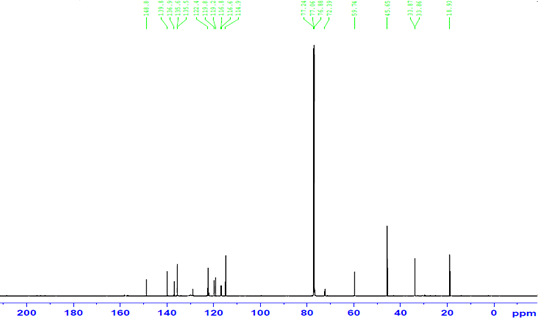


**(B)**

Supplement: S3 Fig — 13C-NMR spectra of acetohydrazide derivative of CZT (A) and that of the standard CZT (B). (DOCX) [file pone.0212048.s003.docx]

**S4 Figure**


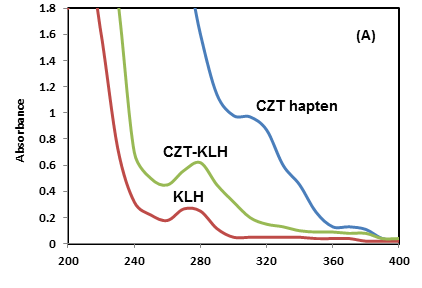

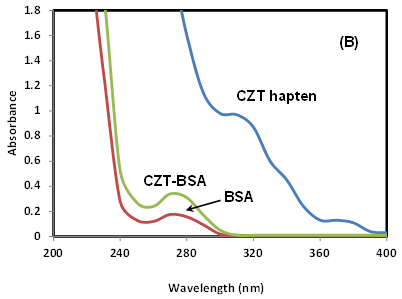

Supplement: S4 Fig — UV-absorption spectral analysis for CZT-protein conjugates with KLH (A) and BSA (B). In panel A: concentrations were 0.1, 1.6, and 1.6 mg/mL for CZT hapten, KLH, and CZT-KLH, respectively. In panel B: concentrations were 0.1, 0.6, and 0.6 mg/mL for CZT hapten, BSA, and CZT-BSA, respectively. (DOCX) [file pone.0212048.s004.docx]
